# Evaluation of quality of life among dental students using WHOQOL-BREF questionnaire in Saudi Arabia: A cross sectional study

**DOI:** 10.12669/pjms.35.3.213

**Published:** 2019

**Authors:** Nouf Al-Shibani, Reem Al-Kattan

**Affiliations:** 1 *Nouf Al-Shibani, Assistant Professor, Department of Periodontics and Community Dentistry, College of Dentistry, King Saud University, Riyadh, Saudi Arabia*; 2 *Reem Al-Kattan, Assistant Professor, Department of Periodontics and Community Dentistry, College of Dentistry, King Saud University, Riyadh, Saudi Arabia*

**Keywords:** OHRQOL (Oral Health-related Quality of Life), Dental students, Health domains, well-being

## Abstract

**Objective:**

The objective was to use modified version of WHOQOL-BREF to assess the well-being and QOL of dental students of King Saud University based on four major domains.

**Methods:**

The questionnaire related to the survey was distributed to all dental students (N= 782) who were enrolled from 1st year to 5th year in College of Dentistry, King Saud University in the fall of 2018. The questionnaire comprised of four domains having different set of questions i.e. Physical domain, Psychological domain, Environmental domain and Social relationship domain. Two stand-alone questions related to (Overall Quality of life and Satisfaction with health) were also part of the WHOQOL-BREF questionnaire. Cronbach’s Alpha was used to assess the reliability of the WHOQOL-BREF domains. Paired t-tests were conducted to compare the means of the four domains and chi-square. Chi-square test was used to find association of demographic characteristics with four domains and two stand-alone questions.

**Results:**

The overall quality of life and satisfaction with health of the dental students was found to be satisfactorily favorable with environmental domain and moderately favorable with social relationship and physical health domains. Physical health domain with psychological domain was statistically significant (p-value <0.001) whereas physical health domain with social relationship and environmental domain was also found to be statistically significant (p-value <0.001). Respondents, who highly rated their overall quality of life and satisfaction with health, had higher domain scores.

**Conclusion:**

In the present study, overall quality of life and satisfaction with health of dental students in King Saud University was found to be satisfactorily favorable.

## INTRODUCTION

A sense of wellbeing, social functioning and emotions, all are related to Quality of life (QOL). The concept of QOL is so multidimensional, multicultural and complex that no universally accepted definition has been established yet.^1^,^2^ A salutogenic theory of QOL is dependent on four dimensions: interpersonal, global, individual’s personal resources and external.^3^ The Quality of life of health professionals is found to be of concern among researchers, educationists and academicians as there are studies which reveal high levels of stress, anxiety and burnout depressions among health care professionals when compared with general populace.^4^-^6^ Interestingly, evidence also points at dental students experiencing more stress and anxiety levels compared to medical care students.^7^ Dental education communities have raised their concerns on the traditional and conventional training delivery model.^8^ The dental learning environment is seen to be unsatisfactory from student perspective and limited opportunities are available for teachers or students to modify the learning environment.^9^

Multiple factors are associated for high stress levels, burnouts and reduced personal achievement among dental students. These may comprise of demanding curriculum, fewer leisure time, amount of assignments, strained relationships, deadlines and new ethical challenges.^10^,^11^ All these factors may contribute to academic difficulties having unfavourable effect on QOL of dental students.^10^,^12^ A systematic review by Elani et al., explores that dental students experience considerable amount of stress in dental schools due to demanding nature of the curriculum resulting in mood swings, behaviour change, reduced capacity to concentrate, suicidal ideation and thoughts related dropping of school.^6^ Surely, these stressors without suitable coping strategies may contribute to poor QOL and compromise the academic performance of dental students.^13^,^14^

Due to high prevalence of stress, anxiety pressure among dental students there is an increased attention to address their happiness, wellness and well-being.^15^ There are many studies which address the stress dental students undergo during dental programme.^10^,^11^,^16^ Assessment of QOL of dental students may give an insight on their approach towards life, health and other related factors. To our knowledge, from indexed literature there are limited studies on QOL of dental students and other multifaceted factors that may have an influence on the quality of life among students in Saudi Arabia. Therefore, the aim of the present study was to use modified version of WHOQOL-BREF^17^ to assess the well-being and QOL of dental students of King Saud University based on four major domains: physical, psychological, social and environmental.

## METHODS

The Ethical committee of King Saud University approved the study with reference number 5847-2. The questionnaire related to the survey was distributed in classrooms to all dental students (N= 782) who were enrolled from 1st year to 5th year in College of Dentistry, King Saud University in the fall of 2018. All the respondents were assured about the confidentiality of personal information. All surveys were distributed in one week to all the students from 1^st^ to 5^th^ year during their classroom lectures.

In the present study a modified version of WHOQOL-BREF^17^ survey consisting of 24 questions was used to evaluate quality of life across different settings and culture. The questionnaire comprised of four domains having different set of questions i.e. Physical domain (seven items), Psychological domain (six items), Environmental domain (eight items) and Social relationship domain (three items). Two stand-alone questions related to (Overall Quality of life and Satisfaction with health) were also part of the WHOQOL-BREF questionnaire. Each question was rated on Lickert scale (1-5) with higher scores signifying better quality of life. Sociodemographic details of the respondents i.e. dental school year, Sex and marital status were also noted to assist as independent variables.

Responses were composed by means of the professional and encrypted version of Survey Monkey (Palo Alto, CA, USA). All the statistical analysis was performed using statistical package for social sciences (SPSS version 21, Inc., Chicago, US). Descriptive analysis was used to express categorical data as percentage and continuous variables as the mean and standard deviation (SD). Cronbach’s Alpha was used to assess the reliability of the WHOQOL-BREF domains. In addition, paired t-tests were conducted to compare the means of the four domains. Correlation coefficients were computed between the four domains and the two stand-alone questions on overall quality of life and satisfaction with health. Finally, Chi-square test was used to find association of demographic characteristics with four domains and two stand-alone questions.

## RESULTS

A total of 533 participants took part in this cross-sectional survey with an overall response rate of 68.15%. More than half of the participants in the present study were male 277 (52%) and more than three-fourth of the respondents 526 (98.7%) were single. The maximum number of participants involved in the study were third year students (D3) 118 (22.1%) and the minimum number of responses were from 2^nd^ year dental students (D2) 90 (16.90%). (Table-I)

**Table-I:** Descriptive statistics of variables (n=533).

Characteristics	Number	Percentage
*Gender*		
male	277	52.00%
female	256	48.00%
*Dental school year*		
First (D1)	110	20.60%
Second (D2)	90	16.90%
Third (D3)	118	22.10%
Fourth (D4)	111	20.80%
Fifth (D5)	104	19.50%
*Marital status*		
single	526	98.70%
married	7	1.30%
living as married	-	-
separated	-	-
divorced	-	-
widowed	-	-

A summary of descriptive statistics and Cronbach’s alpha for the WHOQOL-BREF domains and two separated items Overall Quality of Life & Satisfaction with health was assessed in the study and are shown in Table-II. The responding students rated overall quality of life and satisfaction with health to be satisfactorily favorable with environmental domain and moderately favorable with social relationship and physical health domains. The mean overall quality of life rating was 3.97 on a scale of 1-5, signifying a good assessment. Similarly, the mean rating on satisfaction with health was slightly lower at 3.78, signifying that the students felt between neutral and good about their health.

**Table-II:** Participants scores on WHOQOL-BREF domains and on quality of life and satisfaction with health items.

Domain / Item	Mean	Std.Dev	Minimum	Maximum	Cronbach’s Alpha
Physical health domain	19.77	3.74	8	35	0.565
Psychological domain	17.64	3.04	6	24	0.360
Social Relationships	7.50	1.70	2	10	0.641
Environment	33.72	5.24	15	45	0.770
Overall quality of life	3.97	0.93	0	5	-
Satisfaction with health	3.78	1.05	1	5	-

Cronbach’s alpha was used to assess the reliability of the WHOQOL-BREF and the four domains, with values greater than or equal to 0.70 denoting satisfactory reliability. Social relationship showed moderately good reliability having Cronbach’s Alpha score of 0.64. Whereas, environmental domain displayed Cronbach’s score of 0.77 displaying satisfactory reliability. Furthermore, for physical domain, Cronbach’s score was 0.56 signifying good reliability. However, fair reliability was observed in psychological domain 0.36. (Table-II).

In addition, paired t-tests were conducted to compare the means of four domains. Physical health domain with psychological domain was statistically significant (p-value <0.001) whereas physical health domain with social relationship and environmental domain was also found to be statistically significant (p-value <0.001). However, mean difference of social relationship with environmental domain was higher than mean difference of psychological and environmental domain. (Table-III).

**Table-III:** Differences between pairs of four domain scores for participants.

Domain / Item	Mean Difference	Std.Dev	t	95% CI	p-value
Physical health and Psychological	2.12	0.20	10.17	(1.71,2.53)	< 0.001
Physical health and Social Relationships	12.27	0.17	68.82	(11.92,12.62)	<0.001
Physical health and Environment	13.95	0.27	49.97	(14.50, 13.41)	<0.001
Psychological and Social Relationships	10.14	0.15	67.15	(9.84,10.43)	<0.001
Psychological and Environment	16.08	0.26	61.22	(16.60, 15.57)	<0.001
Social relationship and Environment	26.22	0.23	109.76	(26.69, 25.76)	<0.001

Correlation coefficients were computed between the four domains and the two stand-alone questions on overall quality of life and satisfaction with health. These correlations ranged from 0.245 to 0.542, and the differences were statistically significant (p<0.01). Respondents who highly rated their overall quality of life and satisfaction with health, had higher domain scores. The correlation between the two stand-alone questions was also statistically significant (p<0.001): a higher overall quality of life rating was observed with Psychological Domain (r=0.542). (Table-IV)

**Table-IV:** Correlations among the four domains and two overall questions.

Domain of Life/ Item	Overall Quality with Health	Overall Satisfaction Health	Physical	Psychological	Social Relationships
Overall quality of life	-	-	-	-	-
Overall satisfaction with health	0.395	-	-	-	-
Physical health domain	0.409	0.271	-	-	-
Psychological domain	0.542	0.444	0.519	-	-
Social relationship domain	0.406	0.245	0.341	0.493	-
Environmental domain	0.493	0.370	0.395	0.542	0.490

***Note***: All correlations were statistically significant at p<0.01

### Association with Demographic Variables

Association of demographic characteristics with overall quality of life & satisfaction with health was assessed in the present study. Overall quality of life and satisfaction with health was not significantly (p > 0.05) associated with demographic characteristics. However, psychological domain was statistically associated (p-value < 0.05) with all three demographic variables. No association of demographic characteristics was observed with other three domains.

## DISCUSSION

The present survey provides a unique assessment of QOL of dental students at College of Dentistry, King Saud University using WHOQOL-BREF questionnaire. The type of survey was distinctive, as it assesses the impact of stress on the oral health quality of life of dental students in an institute in Saudi Arabia.

The overall quality of life of the students in the current study was considerably favourable and their satisfaction with health was between neutral and good. Dental students who rated high quality of life and satisfaction with health were found to have high domain scores as well. The current study displayed an improvement of QOL of students as they progressed from first to fifth year. These results were in harmony with a study by Andre et al., who didn’t express any decline in QOL among dental students among the course of four years.^14^ However, a study by Chazan et al., in Portugal among medical students showed decrease QOL with progressing years.^18^

In the current study the physical health domain mean scores [19.77(3.74)] were comparable to psychological domain mean scores [17.64 (3.04)]. Whereas, the environmental domain means scores was found to be highest [33.72(5.24)]. These results indicate that these domains have had a greater impact on dental students’ quality of life. A study by Andre et al., displayed physical health and social relationship to be dominant component in QOL.^14^ Similarly, a study by Zhang et al., showed psychological and social relationship domain to be influential in medical students. In the authors opinion, the heterogeneity in the results of domains influencing QOL in different and present study is due to the multicultural, multidimensional and complex nature of the concept itself.^19^

Compared to the male, female students exhibited high scores in environmental domain and social relationship domain. These results were in concurrence with studies by Andre et al.,^14^ and Zhang et al.^19^ There was no significant difference in quality of life among both genders. Social relationship adheres to, support from friends, personal relationship and sexual activity.^20^ Since females are more expressive, emotional and sensitive they form deeper social connection with others, which gives them satisfaction in social life and relationships.^20^,^21^ However, studies by Shareef et al., and Muirhead et al., have identified that females are more prone to stress and tend to take more pressure.^21^,^22^ The physical health domain scores and psychological domain scores in the present study were less in females compared to males. This finding was in line with a study by Shareef et al.^21^

Among the dental students in the present study there is a gradual increase in quality of life, psychological scores and physical health domain scores with progressing years. Physical health domain consisted of sleep, work capacity and sleep activity in daily lives. Authors believe that the upward trend seen in physical domain with progressing years is due to a shift to comprehensive patient care and more clinical experiences which is practiced more often in College of Dentistry, King Saud University in 4^th^ and 5^th^ year compared to previous years. Furthermore, psychological domain adheres to enjoyment of life, self-esteem, bodily image, which also showed improvement with advancing years. This type of escalating trend in psychological domain and quality of life scores speculated by authors can be due to a shift of didactic curriculum to more patient focused clinical curriculum and move from assessment-based competencies to competencies which are assessed on patient experiences.

Interestingly, in the present study married students displayed high scores in all domains and high satisfaction with QOL and health compared to single students. This trend can be the outcome of social maturity, companionship and spousal support which occur as a result of marriage. A study by Henning et al., showed low distress in married medical students.^23^ Similarly, another cross-sectional study by Lloyd and Musser exhibited mean stress scores to be minimum in married medical students compared to non-married.^24^

The study has limitations based on its small sample size and study design. A similar larger scale study with increased sample size and representation of all dental colleges in the city may give a better understanding on factors having influence on QOL of dental students. Further, since our study was a cross-sectional design it did not follow the progress of cohort students from first to fifth year. Subsequently, QOL is multi ethnic and multi-cultural and other demographic variables should be accounted for in the future studies i.e. ethnicity, socioeconomic status, race, family business and age. In addition, it is recommended that further studies should be focused to evaluate the diverse concept of quality of life among dental students and practitioners by probing the WHOQOL-BREF domain scores.

## CONCLUSION

Overall quality of life and satisfaction with health was found to be satisfactorily favorable with environmental domain and moderately favorable with social relationship and physical health domains. Surprisingly, a gradual increase in quality of life, psychological scores and physical health domain scores were found in dental students with progressing years. Married dental students displayed high scores in all domains and high satisfaction with QOL and health compared to single students. Encouraging better QOL of dental students is not only important for dental schools, but has a massive impact on dental profession as a whole and translates this into a healthier patient care.
